# Olfactory Reference Syndrome with Suicidal Attempt Treated with Pimozide and Fluvoxamine

**DOI:** 10.1155/2018/7876497

**Published:** 2018-04-15

**Authors:** Oluwole Jegede, Inderpreet Virk, Karthik Cherukupally, Wil Germain, Patricia Fouron, Tolu Olupona, Ayodeji Jolayemi

**Affiliations:** Department of Psychiatry, Interfaith Medical Center, Brooklyn, NY 11213, USA

## Abstract

The core symptomatology of the Olfactory Reference Syndrome (ORS) is characterized by a preoccupation with the belief that one emits an offensive odor, albeit not perceived by others. The present case is that of a 75-year-old African American woman, with an unclear past psychiatric history, who was brought into our Emergency Room after a suicide attempt. The patient reported a three-year history of a “rotten” smell from her vagina. She adamantly believes that she smells despite being told otherwise by people. The patient reported a trial of several feminine products to get rid of this smell and multiple visits to specialists but her symptoms persisted. Her symptoms involved a significant depressed mood and deterioration in her social functioning, interpersonal relationships, and self-care. She was constantly in the shower and had stopped leaving her apartment due to worries that people might smell her vagina. The culmination of her distress was the suicidal attempt, for which she was brought to the hospital. She was admitted to the inpatient psychiatric unit and started on Pimozide and Fluvoxamine. The patient made remarkable progress within a few days on admission and in the course of her hospitalization. Follow-up in our outpatient clinic shows that the patient remains completely asymptomatic with significant progress in her social functioning.

## 1. Introduction

The symptoms of Olfactory Reference Syndrome (ORS) were first described in a case series of 36 patients by Pryse-Phillips in 1971 [1]. Although published literature on the subject spans more than a century, areas of controversies persist in terms of the nosology and treatment of the disease [1]. The core symptomatology of ORS is characterized by a preoccupation with the belief that one emits an offensive odor, which is not perceived by others [1, 2]. Other terms that have been used in literature to describe the disease include delusions of bromosis, hallucinations of smell, chronic olfactory paranoid syndrome, olfactory delusional syndrome, monosymptomatic hypochondriacal psychosis, olfactory delusional state, olfactory hallucinatory state, and autodysomophobia [3].

The characterization of this syndrome has been a moving target; it appears in the DSM 5 under “Other Specified Obsessive-Compulsive Disorders” as well as under the “Glossary of Cultural Concepts of Disease,” as a variant of* Taijin Kyofusho*, a disease characterized by “anxiety about and avoidance of interpersonal situations, due to the thought, feeling, or conviction that one's appearance and actions in social interactions are inadequate or offensive to others.” [4]. ORS was first categorized as an atypical somatoform disorder in the DSM-III and then as a delusional disorder in DSM-IV-TR and now under Other Specified Obsessive-Compulsive Disorders in DSM 5 [2]. The controversy surrounding its classification stems from the supposed preferential response of the condition to Selective Serotonin Reuptake Inhibitors (SSRIs) suggesting a possible associational overlap with Obsessive-Compulsive Spectrum Disorders and its very strong comorbidity with depressive disorders but, despite this preference, reports of the utility of antipsychotics such as Quetiapine, Risperidone, and Pimozide have also been reported in literature [1, 5].

The clinical course of ORS is chronic and debilitating for the patient and their families; although the clinical presentation may be confused with primary psychotic disorder, there is no clear evidence that this disorder leads to or is associated with schizophrenia [1, 6, 7]. Pryse-Phillips, in his seminal paper, highlighted the importance of depression as the most common psychiatric comorbidity with ORS but other comorbidities have also been described in literature including bipolar disorder, personality disorders, schizophrenia, hypochondriasis, alcohol and substance use disorders, Obsessive-Compulsive Disorder (OCD), and body dysmorphic disorder [1, 8].

Unfortunately, literature on ORS is very sparse and research is limited. Available reports are mainly limited to case reports, case series, or literature reviews; as of yet, there are no diagnostic criteria clearly agreed upon that define the syndrome [9, 10]. Few systematic studies have outlined clinical symptoms of ORS, other than the Pryse-Phillips paper; an Internet based study by Greenberg and colleagues describes the complexity of the clinical features; they reported that nearly all participants performed rituals in response to ORS beliefs and endorsed avoidance of usual social and occupational activities including significant avoidance of intimacy, physical activities, and various social situations [1, 10]. These findings are in keeping with previous reports of individuals with ORS who tend to exhibit time-consuming rituals aimed at treating the odor and avoid social situations, and they experience profound impairment in social and occupational functioning as well as suicidal ideation and attempts.

## 2. Case Presentation

We present a case of a 75-year-old African American woman, widow, unemployed, and domiciled with a past medical history of hypertension, osteoarthritis, and asthma. The patient was brought to the Emergency Room by Emergency Medical Services (EMS) on account of an attempted suicide due to a 3-year history of “bad odor coming from my vagina.” The patient reported that the foul smell from her vagina was making her body “rotten.” She reported that “the smell came back recently and it is stronger.” Although she has been having the odor for the last 3 years, it has only recently gotten worse, the culmination of which resulted in her attempted suicide this time. She reported that she has seen several gynecologists who have treated her to no avail and later advised her to see a psychiatrist. She stated that there is a “devil” in her body that does not let go and she said “I need help.” The patient has a significant impairment in social functioning evidenced by a reported avoidance of social events; she could no longer go out to the store for her basic needs; according to the patient's son, she has also stopped going out to get groceries or to the church. She reported that she has been unable to have any romantic relationships because of her “odor.” The patient stays at home all day, showers several times daily, and has tried many vaginal products and creams but all in vain.

At the time of initial evaluation, the patient appeared paranoid, reporting that people stayed away from her because of her smell. She also endorsed ideas of reference claiming that people around her cover their noses, stand next to windows, or look at her in “a certain way” and then talk about how much she “stinks” to each other. She endorses profound feelings of hopelessness, helplessness, and guilt and was tearful during the interview. Other symptoms reported were poor sleep, feeling less energetic, decrease in concentration, and anhedonia. She also endorsed active suicidal ideation, imagining waking up dead every morning due to her odor, and attempted to stab herself in order to “end my mystery” which led to this current admission. She also reported that she had lost up to 20 pounds in last 3 months. The patient was initially diagnosed with schizophrenia but later revised to Olfactory Reference Syndrome (ORS) in view of an extensive review of her symptoms and collateral information.

## 3. Hospital Course

The patient was admitted to the inpatient psychiatric unit and placed on 1 : 1 constant observation for active suicidal ideation. Laboratory investigations including urine toxicology, liver function, urea, creatinine, electrolytes, and antinuclear antibodies, syphilis, and human immunodeficiency virus serology were all within normal limits or negative. She was started on Risperdal 2 mg PO twice daily for psychosis, Escitalopram 20 mg PO daily for depression, and Trazodone 50 mg PO HS for sleep. Neurological and gynecological consults were sought and the MRI of the brain obtained revealed no significant findings and was otherwise unremarkable. After a week, the patient's delusions about her vaginal smell got even worse. She would not go outside of her room even for meals which were offered to her in the room because she thought that people could smell her vaginal odor. She also spent very long hours in the showers and demanded to take showers several times daily; her requests put a strain on the staff of the unit and on other patients who needed to use the same facilities. The patient's medications were reviewed and she was started on Pimozide 1 mg PO twice daily and Fluvoxamine 25 mg PO daily based on the revision of her diagnosis to ORS. Risperdal, Citalopram, and Trazodone were discontinued. The patient made remarkable progress in the next few days. Pimozide was optimized to 2 mg PO twice daily and Fluvoxamine to 75 mg PO daily during the course of her hospitalization. She remained adherent with her medications and no side effects were noted. The patient and nursing staff agreed to a 70% symptomatic improvement in the patient's symptoms; her affect was brighter; she was able to go outside of her room for meals and group therapy and socialize with other patients and staff. She became amenable to dissuasion regarding her previously held delusions and denied any depressive symptoms and no longer needed 1 : 1 constant observation as she was no longer suicidal. She appeared future oriented and motivated to go back home and resume her social life again. She was discharged back to her apartment and was provided with outpatient appointment for aftercare. The team followed up with the patient patients several months after her discharge and she continued to maintain a remission of her symptoms.

## 4. Discussion

Our patient believed that her vagina was emitting such a strong odor that she attempted to take her own life after 3 years of significant distress. Her belief was accompanied by ideas of reference; that is, she thought that other people took special notice of the odor in a negative way; she performed repetitive behaviors of multiple daily showers and use of vaginal washing soaps daily. Although not an official diagnostic criterion, our patient met the provisional criteria set by the DSM-5 Anxiety, Obsessive-Compulsive Spectrum, Posttraumatic, and Dissociative Disorders Work Group criteria for Olfactory Reference Syndrome [2]:

- Preoccupation exists with the belief that one emits a foul or offensive body odor, which is not perceived by others.
- The preoccupation causes clinically significant distress (e.g., depressed mood, anxiety, and shame) or impairment in social, occupational, or other important areas of functioning.
- The symptoms are not a symptom of schizophrenia or another psychotic disorder and are not owing to the direct physiological effects of a substance (e.g., drug abuse or medication) or a general medical condition.

The comorbidity with Major Depressive Disorder in our patient is of particular significance. The importance of this comorbidity is well known and has been reported in literature [1]. In this case, our patient reported several symptoms suggestive of Major Depressive Disorder evidenced by her profound feeling of hopelessness and guilt; she has lost interest in everything; she reported insomnia and poor appetite with significant amount of weight loss. All the patient's symptoms, although rooted in context of her perception that she was smelling, were nonetheless significant to the point that she attempted suicide.

The use of Pimozide and SSRIs in the treatment of monosymptomatic hypochondriacal states has been consistently reported in literature [11, 12]. The combination of these medications in the index case yielded excellent results. Although the reliability of the diagnostic criteria is not yet established and ORS is not a stand-alone diagnosis in the DSM-5, it merits consideration in patients who present with monosymptomatic hypochondriacal illnesses, as this diagnostic consideration may influence the treatment and eventually the potential course of the illness as with our patient who after three years of a distressing illness is currently in remission with proper treatment.
